# Pellagra Forum

**Published:** 1916-07

**Authors:** 

**Affiliations:** Hot Springs, Ark.


### PELLAGRA FORUM.

DR. E. H. MARTIN, EDITOR, HOT SPRINGS, ARK.

Address all communications on this subject to Dr. Martin.

The editor of the TEXAS MEDICAL JOURNAL has decided to devote a portion of the space in the JOURNAL to pellagra. This is a very wise decision, for there is no reader of the JOURNAL who is not vitally interested in that disease. The object of this department is to publish papers on the subject and also to invite criticism and discussion of these papers and of the subject in general.

In accepting the position of "Pellagra Editor," it is with the intention of giving every opinion and theory a hearing. Although my own opinions have been published and my theories, as to treatment at least, have been backed by spectacular cures in a large percentage of cases, and in the hands of others also, I have also had a sufficient number of dismal failures to make me take interest in the several other successful "cures" so honestly believed in by able men.

I have yet to read an enthusiastic paper heralding a successful treatment of pellagra which does not give a larger percentage of cures than I have been able to obtain with what seems to me to be a direct specific in a large proportion of cases. I know that the men who report a miraculous percentage of cures from diet or quinine or alkalies or picric acid or autogenous serum or santonin are all honest and should have a hearing.

It is also evident that a disease which has so many absolute and reliable "cures" and which still provides such a large death rate in all of the Southern States is a proper subject for discussion.

As the able editor of this JOURNAL has suggested to me, some of the greatest discoveries in the medical world have been made or suggested by unknown men. Therefore, there is reason why every practitioner should voice his opinion and tell of his observations. If it should happen that the "Pellagra Editor" sees fit to make light of such opinion, solace may be had by the contributor in remembering how the profession of London ridiculed Jenner and how the great Harvey was made unhappy because he saw the truth and knew it when he saw it.

So we wish and invite free expression of opinions and details of observations from all those who feel that they have a message, whether from laboratory or saddle-bags.

E. H. MARTIN.

---

